# A Sharpness-Optimized Partitioned PSF Estimation Method for UAV TDI Push-Broom Image Deblurring

**DOI:** 10.3390/s26082414

**Published:** 2026-04-15

**Authors:** Zhen Zhang, Min Xu

**Affiliations:** Shanghai Engineering Research Center of Ultra-Precision Optical Manufacturing, College of Future Information Technology, Fudan University, Shanghai 200433, China

**Keywords:** image deblurring, time delay integration, UAV, sharpness optimization

## Abstract

**Highlights:**

**What are the main findings?**

- Established a coupled degradation model for UAV TDI imaging. This physical model accurately characterizes the joint impact of platform vibration and velocity mismatch during the multi-stage charge accumulation process.
- Enhanced imaging quality in low-light environments.

**What are the implications of the main findings?**

- Developed a partitioned PSF estimation algorithm based on sharpness optimization. For the first time, partitioned PSF estimation is integrated with image sharpness criteria to achieve adaptive iterative search and improves the image quality of the blurred image.

**Abstract:**

In uncrewed aerial vehicle (UAV)-based ground observation and detection missions involving high-speed moving targets or low-light conditions, Time Delay Integration (TDI) cameras enhance image brightness through multi-stage charge accumulation. However, the imaging quality is susceptible to motion blur induced by platform vibrations and velocity mismatch. Based on TDI imaging technology, a TDI image degradation model for a UAV-based imaging platform is formulated. To address spatial blurring caused by platform vibration and velocity mismatch during TDI imaging, we propose a TDI image restoration algorithm based on sharpness-optimized partitioned Point Spread Function (PSF) estimation. The main innovation lies in the first application of partitioned PSF estimation combined with image sharpness optimization in TDI imaging. By formulating an accurate TDI image degradation model, spatial motion blur kernel estimation is transformed into an iterative search problem for partitioned optimal PSF. Solving for optimal sharpness yields the optimal PSF and corresponding local motion parameters, achieving image restoration. Simulation and experimental results demonstrate that the proposed algorithm in this paper effectively removes motion blur in TDI dynamic imaging, while suppressing artifacts and ringing, thus significantly enhancing image quality.

## 1. Introduction

In recent years, low-altitude, high-speed ground observation technology based on uncrewed aerial vehicles (UAVs) has rapidly become a global research focus [1,2,3]. Owing to their simple structure, light weight, and low manufacturing and operating costs, UAVs offer significant advantages across a wide range of application domains. They demonstrate irreplaceable capabilities, particularly when executing missions that are considered dangerous or unsuitable for human pilots. Meanwhile, their high flexibility and multifunctionality enable effective deployment in diverse, complex, and extreme environments, providing critical data and information support. Consequently, UAVs are widely applied in numerous domains, including but not limited to military reconnaissance and intelligence gathering, natural disaster monitoring and emergency response, dynamic monitoring and protection of ecological environments, radiation and pollution monitoring in industrial sectors, crop growth monitoring in precision agriculture, and safety inspections of buildings and infrastructure [4,5,6,7]. As the demand for high-resolution, low-light, and high-speed imaging continues to grow in application scenarios such as nighttime flight, as well as operations under rain, fog, and overcast conditions, obtaining high-quality images under low-light dynamic flight conditions has become an urgent challenge.

For low-light imaging, current research primarily focuses on the following technologies: low-light night vision imaging, infrared imaging, laser active illumination imaging, and computational image enhancement [8,9,10,11]. Among them, infrared imaging has limited resolution and can suffer from image distortion, laser active illumination is susceptible to scattering interference and may expose targets, while computational enhancement often faces issues such as high computational complexity and insufficient real-time processing capability, and it is prone to introducing noise and artifacts when parameters are improperly estimated or the model overfits [11]. Low-light night vision imaging leverages the photoelectric conversion and image intensification capabilities of detectors such as Complementary Metal Oxide Semiconductor (CMOS), Charge Coupled Device (CCD), and image intensifiers to amplify target signal strength in low-light environments, enabling the detection of minute targets under conditions relying solely on ambient light. Compared to conventional CCD and CMOS sensors, Time Delay Integration (TDI) cameras offer a high signal-to-noise ratio (SNR) and exceptional low-light imaging capabilities [12]. Their high dynamic range (HDR) characteristics enable dynamic adjustment of integration stages, effectively adapting to brightness variations within scenes. This prevents overexposure and underexposure, ensuring precise capture of image details. The low-light imaging and high dynamic range properties of TDI cameras make them particularly well-suited for imaging tasks in low-illumination environments and high-speed dynamic scenes.

TDI cameras capture image information using line scanning technology, which requires extended exposure times. This makes them highly sensitive to abnormal motion disturbances. If a target moves abnormally during exposure, it not only causes image blur but also induces geometric distortion, significantly degrading image quality. During actual UAV flight operations, imaging quality is adversely affected by variations in platform attitude, irregular mechanical vibrations of the camera, relative motion between the camera and the target, atmospheric disturbances, and platform vibration. These factors cause captured aerial images to exhibit complex noise, blur, low contrast, and loss of fine texture, leading to geometric distortion and image blur [13,14]. Several methods have been proposed to compensate for image distortion caused by platform vibration and velocity mismatch. Tong et al. [15] corrected attitude-induced vibration distortion in high-resolution satellite images through image resampling and attitude data compensation. The image resampling process used cubic convolution interpolation, while attitude data compensation adjusted the CCD field of view to eliminate vibration effects. Pan et al. [16] proposed an intelligent micro-misalignment push-broom imaging architecture that introduced minute offsets in the orthogonal direction of the TDI array, leveraging the satellite’s intrinsic motion to achieve multi-frame sub-pixel phase shift sampling. Finally, a sparsity-regularized computational reconstruction algorithm was applied to restore high-resolution information. Guo et al. [17] analyzed UAV attitude and vibration data using inertial measurement unit (IMU) information to compute image Point Spread Function (PSF). They used total variation (TV) regularization to restore blurred images. Hayashi et al. [18] estimated motion parameters of the blur kernel from image gradient information, determining the direction and extent of the blurred PSF. They then employed non-blind deconvolution algorithms to recover image sharpness.

However, these methods typically rely on additional attitude information and extremely high platform stability. Although some studies have applied related techniques to conventional camera imaging or vibration-induced blur in TDI systems, the unique and dynamic motion characteristics of UAV platforms have not been fully considered, making these methods difficult to directly apply to UAV-based TDI imaging under complex flight conditions. Accordingly, this paper proposes a partitioned deblurring algorithm grounded in the principle of optimal sharpness. By formulating a motion-degradation model for TDI images, it accurately estimates and iteratively refines the spatially varying blur kernel induced by platform vibration and velocity mismatch, thereby achieving high-quality restoration of TDI images.

This paper first introduces the imaging principle of TDI images and constructs a UAV-based TDI image degradation model by considering the characteristics of TDI imaging. On this basis, a TDI image restoration algorithm based on sharpness-optimized regional PSF estimation is proposed. Subsequently, the effectiveness of the proposed algorithm in this paper is validated through both numerical simulation and real-world experimental results. Finally, the research work is summarized, and its potential applications are discussed.

## 2. Principle

### 2.1. TDI Imaging Principle

TDI technology is a high-sensitivity, high-SNR push-broom imaging technique that is widely used in aerospace remote sensing, industrial non-destructive testing, and medical imaging. Unlike traditional area-scan cameras, TDI image sensors are specialized line-scan devices featuring a linear array architecture with area-scan output capability. The core mechanism of this technology involves precisely synchronizing the continuous transfer of sensor charge with platform motion during push-broom imaging. This enables multiple rows of pixels to accumulate exposure from the same target, completing data acquisition in line-scan mode [19].

**Figure 1:**
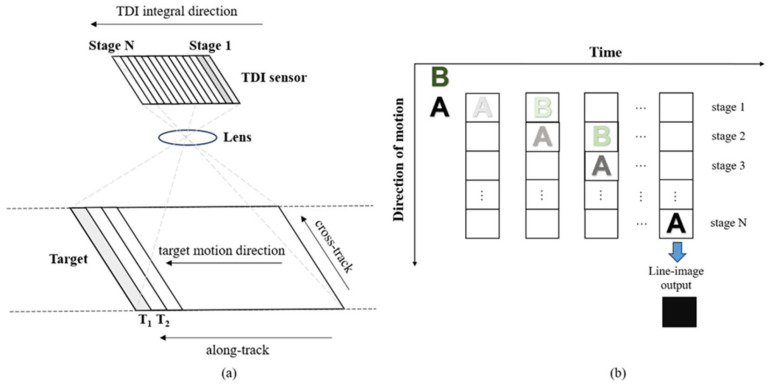
Imaging Principle of TDI Image Sensor: (**a**) TDI operating mode; (**b**) Charge accumulation characteristics of TDI per line.

The imaging principle diagram of the TDI image sensor is illustrated in Figure 1. Figure 1a illustrates the operation of the TDI image sensor: when the target moves left along the track, the integration direction of the TDI image sensor is also leftward. As the target moves, the TDI sequentially exposes and accumulates signals at the same object position from stage 1 to stage N. Specifically, stage 1 first exposes the target at position T_1_. As the target moves leftward, when stage 2 exposes position T_1_, stage 1 simultaneously exposes position T_2_. Through this stage-by-stage charge transfer and cumulative signal integration process, the target along the track is effectively captured. Figure 1b illustrates the stage-by-stage charge accumulation characteristic during TDI imaging: when the target moves, stage 1 exposes the first row of the target, generating initial photoelectric charge. After one TDI cycle (row integration time), the charge from stage 1 transfers to stage 2. Simultaneously, stage 1 and stage 2 expose the second row and first row of the target, respectively, generating new photoelectric charge. With each TDI cycle, every stage in the TDI sensor simultaneously integrates newly incident photon energy while receiving transferred charge from the preceding stage, thereby achieving cumulative signal enhancement. When the charge reaches the final row of pixels, the accumulated signal is read out to form the final image signal.

Based on the charge accumulation principle of TDI image sensors, for a TDI sensor with N integration stages, the same object undergoes N exposures and accumulations, and the signal S from a single image point is linearly summed over the N stages [19]. If the signal generated by a single (i-th) exposure is $S_{i}$, then the total output signal intensity $S_{TDI}$ can be expressed as follows:

(1) $$S_{TDI}=\sum_{i=1}^{N}S_{i}=N\cdot S$$

The primary noise sources in TDI image sensors are independent and random, following a Poisson distribution. If the standard deviation of noise generated by a single exposure is σ, then the total noise $\sigma_{TDI}$ can be expressed as follows:

(2) $$\sigma_{TDI}=\sqrt{\sum_{i=1}^{N}\sigma^{2}}=\sqrt{N}\cdot \sigma$$

The $SNR_{TDI}$ of a TDI image sensor can be expressed as follows [20]:

(3) $$SNR_{TDI}=\frac{S_{TDI}}{\sigma_{TDI}}=\frac{N\cdot S}{\sqrt{N}\cdot \sigma }=\sqrt{N}\cdot \frac{S}{\sigma }=\sqrt{N}\cdot SNR$$

According to Equation (3), the $SNR_{TDI}$ of the TDI image sensor is $\sqrt{N}$ times higher than that of a conventional area-scan camera with a single exposure. This enables the TDI image sensor to effectively address the challenges of low-light environments and acquire images with a higher SNR.

### 2.2. Velocity Matching Principle

In scenarios where UAVs operate at low altitudes and high speeds, objects on the ground are projected onto the focal plane through the image optical system. When the UAV platform flies at an average velocity V along the scanning direction of the TDI image sensor, ignoring terrain undulations and aberrations, the movement velocity $v_{o}$ of the image point P’ on the focal plane corresponding to a ground point P can be expressed as follows [21]:

(4) $$v_{o}=V\cdot \frac{f}{H}$$

where H denotes the height between the UAV and the object, and f denotes the focal length of the optical system.

The TDI image sensors accumulate charges stage by stage through multiple exposures of the same object. To ensure imaging quality in high-dynamic environments, the charge transfer time between adjacent TDI stages must match the image motion velocity, which is expressed by the TDI velocity matching formula; only under this condition can the TDI sensor produce sharp images [22]. The TDI velocity matching formula requires that the charge transfer speed of the TDI sensor be consistent with the motion speed of the target image on the focal plane, in order to avoid severe motion blur. The relationship between charge transfer time T and image shift velocity $v_{o}$ can be expressed as follows:

(5) $$\begin{array}{ll} p= & v_{o}\cdot T\\ T= & \frac{p}{v_{o}}=\frac{p}{V}\cdot \frac{H}{f} \end{array}$$

where p is the pixel size of the TDI image sensor, and the line scan frequency $f_{line}=1/T$.

During the imaging process of a TDI image sensor, based on its multi-stage charge accumulation characteristics, the accumulated charge on the TDI CCD can be expressed as the sum of the charge transferred from the previous TDI stage and the charge generated in the current stage. Its mathematical model is expressed as:

(6) $$\begin{array}{ll} g(i,j,k) & =g(i-1,j,k-1)+s(i,j,k)\\ s(i,j,k) & =Kq\int_{(k-1)T}^{kT}\iint_{A_{ij}}\varepsilon (x,y,t)dxdydt \end{array}$$

where g(i,j,k) denotes the accumulated charge at position (i,j) on the TDI CCD after k integration cycles, with i and j representing rows and columns respectively on the TDI CCD; s(i,j,k) denotes the newly generated charge at position (i,j) during the kth integration cycle; K denotes the system gain of the readout electronics, which converts accumulated charge into the digital output signal; q is the elementary charge of a single electron; $A_{ij}$ denotes the effective light-sensitive area of the (i,j)-th pixel in the sensor, which is also denotes the area occupied by a pixel (p×p), and ε(x,y,t) denotes the electron generation rate per unit area per unit time at the continuous spatial position (x,y).

**Figure 2:**
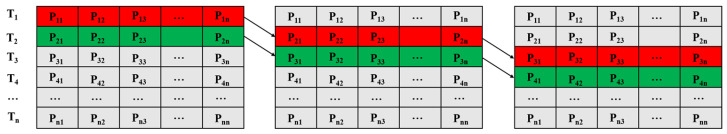
Charge accumulation of TDI per line.

Ideally, the final output image $f_{d}\left(u,v\right)$ can be expressed as follows:

(7) $$f_{d}\left(u,v\right)=q\sum_{k=1}^{N}\int_{(k-1)T}^{kT}\iint_{A_{ij}}\varepsilon (x,y,t)\delta (u-i+k-1)\delta (v-j)dxdydt$$

where $\left(u,v\right)$ denotes the pixel position. δ(u−i+k−1) represents the row-coordinate constraint, and δ(v−j) represents the column-coordinate constraint. These constraints describe the mapping relationship between the CCD pixel coordinates and the final output pixel. Only the charges that satisfy this mapping relationship can be accumulated into $\left(u,v\right)$. The detailed charge accumulation process is illustrated in Figure 2.

### 2.3. TDI Image Degradation Model

However, during actual UAV flight imaging operations, the performance of TDI image sensors is constrained by multiple degradation factors. On one hand, factors such as platform-induced vibration from the UAV itself and atmospheric transmission effects degrade the imaging stability of the TDI image sensor. On the other hand, TDI imaging requires relatively long exposure times, making it highly sensitive to abnormal motion. Any unexpected target movement during exposure not only introduces motion blur but also leads to geometric distortion. Therefore, it is necessary to construct a non-ideal imaging mathematical model to accurately describe the degradation process of TDI images.

The degradation model for TDI images can be constructed using a blur function and an additive noise term. The degradation process can be expressed as follows [23]:

(8) g(x,y)=h(x,y)∗f(x,y)+n(x,y)

where g(x,y) denotes the blurred image, h(x,y) denotes the blur kernel, which corresponds to the PSF term, f(x,y) denotes the sharp image, n(x,y) denotes the additive Gaussian noise, and ∗ denotes the 2D convolution.

**Figure 3:**
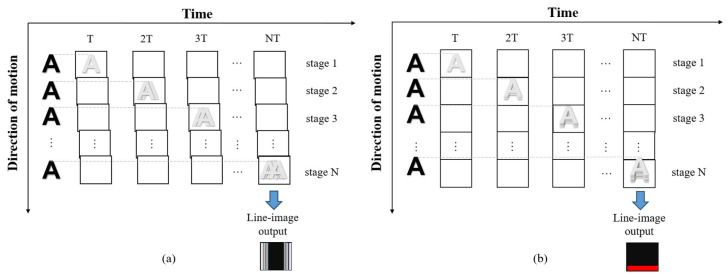
TDI Operation Process Under Non-Ideal Conditions: (**a**) When platform vibration from the UAV itself introduces vibration, the final TDI image becomes blurred; (**b**) When the charge transfer time does not match the UAV’s flight speed, the final TDI image becomes blurred.

TDI blurring differs from conventional image blurring processes. Figure 3 shows the TDI operation under non-ideal conditions, illustrating the TDI blurring process. When factors that degrade TDI images, including vibration or velocity mismatch, are present, they cause relative shifts in the positions of image points formed by the same target across different TDI stages. Due to TDI’s unique characteristic of charge accumulation across successive stages, the relative image shift error generated between adjacent stages affects not only the next stage but all subsequent stages as well.

The blur kernel for the final output pixel is determined by the image shift deviation value $\Delta r\left(t\right)=\left(\Delta x\left(t\right),\Delta y\left(t\right)\right)$. Here, $\Delta x\left(t\right)$ represents the deviation value along the scanning direction, while $\Delta y\left(t\right)$ denotes the deviation value perpendicular to the push-pull scanning direction. In the TDI imaging process, due to the very high line frequency, the time interval between adjacent lines is extremely short, on the order of microseconds. Within such a brief observation period, the relative velocity difference caused by UAV platform vibration or velocity mismatch can be approximated as piecewise constant. $\Delta x\left(t\right)$ and $\Delta y\left(t\right)$ can be expressed as follows:

(9) $$\left\{\begin{array}{l} \Delta x\left(t\right)=\left(v_{o}-v_{scan}\right)\cdot T+A_{x}\sin(\omega_{x}t+\phi_{x})\\ \Delta y\left(t\right)=A_{y}\sin(\omega_{y}t+\phi_{y}) \end{array}\right.$$

where $v_{scan}$ denotes the interline charge transfer velocity, $A_{x}$ and $A_{y}$ denote the vibration amplitudes in the x and y directions respectively, $\omega_{x}$ and $\omega_{y}$ denote the vibration angular frequencies in the x and y directions respectively, and $\phi_{x}$ and $\phi_{y}$ denote the initial phases in the x and y directions respectively.

After introducing the image shift deviation value, the newly generated charge on the TDI sensor during the kth integration cycle is:

(10) $$s(i,j,k)=q\int_{(k-1)T}^{kT}\iint_{A_{ij}}\varepsilon (x-\Delta x(t),y-\Delta y(t),t)dxdydt$$

where ε(x−Δx(t),y−Δy(t),t) denotes the electron generation rate per unit area per unit time after introducing the image shift deviation.

The final output pixel h(u,v) can be expressed as follows:

(11) $$h(u,v)=q\sum_{k=1}^{N}\int_{(k-1)T}^{kT}\iint_{A_{ij}}\varepsilon (x-\Delta x(t),y-\Delta y(t),t)\delta (u-i+k-1)\delta (v-j)dxdydt$$

Based on the aforementioned TDI image degradation model, it can be concluded that after introducing image shift deviation, the blurring of TDI images is primarily caused by image shift deviation in the x-direction, while the influence of image shift deviation in the y-direction is relatively small.

## 3. TDI Image Restoration Method Based on Partition Point Diffusion Function Estimation and Optimal Sharpness Method

### 3.1. TDI Image Restoration Algorithm Based on Partitioned PSF Estimation with Optimal Sharpness

TDI image degradation differs from conventional image blur, as the blur kernel H carries the row-by-row cumulative effect of image shift deviation $\Delta r\left(t\right)$ across N charge transfer cycles. Under the coupled effects of UAV platform vibration and velocity mismatch, the instantaneous image shift errors generated within each charge transfer cycle are progressively amplified and superimposed during the TDI charge transfer process. This results in the blur kernel H within the TDI image degradation model exhibiting pronounced row-by-row variation characteristics.

**Figure 4:**
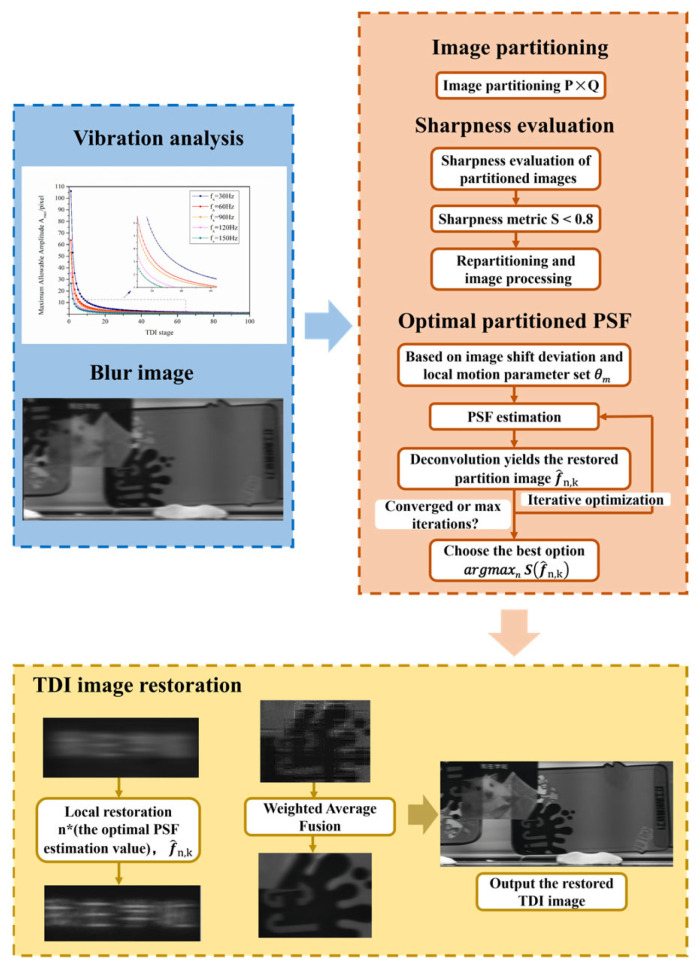
Flowchart of the TDI Image Restoration Method Based on Optimal Sharpness Partitioning PSF Estimation.

The proposed TDI image restoration method based on sharpness-optimized partitioned PSF estimation is illustrated in Figure 4. This method comprises five steps: (1) degraded image input and platform vibration analysis, (2) image partitioning, (3) sharpness threshold evaluation, (4) construction and optimal estimation of partitioned PSFs, and (5) local restoration and stitching fusion.

Analysis of image degradation caused by UAV platform vibration primarily involves determining the maximum vibration amplitude and comparing it with the current UAV stabilization capability to evaluate the influence of platform vibration on TDI imaging performance. Based on this, we applied a sinusoidal excitation to the UAV’s X-axis direction (parallel to the TDI push-broom scanning direction) to simulate platform vibration. When the UAV moves along the TDI scanning direction with a flight velocity of $v_{u}$, the motion function in the x-direction can be expressed as follows:

(12) $$x\left(t\right)=v_{u}t$$

Under these conditions, the light intensity can be expressed as follows:

(13) $$I\left(t\right)=I_{0}+I_{m}\cos(2\pi f_{c}v_{u}t)$$

where $I_{0}$ is the original scene brightness, $I_{m}$ is the amplitude of the sinusoidal brightness, $f_{c}$ is the vibration frequency.

The TDI image sensor accumulates charge through N exposures during imaging, with an equivalent exposure time of NT. By averaging the light intensity over this cycle, the following can be obtained:

(14) $$\bar{I\left(t\right)}=\frac{1}{NT}\int_{0}^{NT}I(t)dt=\frac{1}{NT}\cdot NTI_{0}+\frac{1}{NT}\cdot I_{m}\cdot {\left.\;\left[\frac{\sin\left(2\pi f_{c}v_{u}NT\right)}{2\pi f_{c}v_{u}}\right]\right|}_{0}^{NT}$$

(15) $$\bar{I\left(t\right)}=I_{0}+I_{m}\cdot \frac{\sin\left(2\pi f_{c}v_{u}NT\right)}{2\pi f_{c}v_{u}NT}$$

(16) $$\bar{I\left(t\right)}=I_{0}+I_{m}\cdot \sin c\left(\pi f_{c}v_{u}NT\right)\cos\left(\pi f_{c}v_{u}NT\right)$$

The modulation transfer function (MTF) of a system is defined as the ratio of output modulation $M_{out}$ to input modulation $M_{in}$, and can therefore be expressed as follows [24,25]:

(17) $$M_{in}=\frac{I_{m}}{I_{0}}$$

(18) $$M_{out}=\frac{I_{m}\cdot \sin c\left(\pi f_{c}v_{u}NT\right)}{I_{0}}$$

(19) $$MTF=\frac{M_{out}}{M_{in}}=\;\sin c\left(\pi f_{c}v_{u}NT\right)$$

From the above equation, it follows that the imaging quality of the imaging system is jointly governed by modulated coupled parameters, including the integration stages, the vibration frequency, UAV flight speed, and other factors.

To prevent significant degradation in image quality, image shift should be controlled within one pixel [26]. Based on the preceding formula derivation, the system’s image shift Δx in the x-direction can be expressed as follows:

(20) $$\Delta x=NTv_{u}$$

At this point, Δx should satisfy:

(21) Δx≤ p

Traditional deblurring methods typically employ a globally uniform PSF. However, for blurred images generated by TDI cameras mounted on UAVs during push-broom scanning, the PSF often varies across the entire image due to factors such as varying ground-projection velocities and platform-induced vibrations. Therefore, we divide the input degraded image into P × Q subregions and use the mean gradient magnitude as the fundamental sharpness metric S(f) to evaluate the validity of the partitioning.

(22) $$S\left(f\right)=\sum_{i,j}\sqrt{G_{x}{\left(i,j\right)}^{2}+G_{y}{\left(i,j\right)}^{2}}$$

where $G_{x}$ and $G_{y}$ represent the horizontal and vertical gradients of the partitioned image f, respectively.

When the S(f) value falls below the threshold, it indicates severe blurring or high noise interference in that region, necessitating further subdivision to enhance the accuracy of local PSF modeling. The local blur kernel $h_{m}$ for each partition must be constructed using the TDI image degradation model. The kernel length is directly related to the underlying degradation factors. Along the TDI scan direction, the kernel length can be expressed as follows:

(23) $$L_{x}=\left(v-v_{scan}\right)\cdot t^{\prime }+A_{x}\sin(\omega_{x}t+\phi_{x})$$

Perpendicular to the TDI push-broom direction, the kernel length can be expressed as follows:

(24) $$L_{y}=A_{y}\sin(\omega_{y}t+\phi_{y})$$

The local blur kernel is related to the cumulative projection of the trajectory of instantaneous image plane displacement within the TDI period onto the image plane, i.e., it is related to the image shift deviation value Δr(t):

(25) $$h_{m}(x,y)\propto \sum_{k=1}^{N}\left[\int_{(k-1)T}^{kT}P(\Delta x(t),\Delta y(t))dt\right]$$

where P(Δx(t),Δy(t)) represents the image shift trajectory function, i.e., the position of the instantaneous image point.

From the above equation, the image shift offset is identified as the key factor in constructing the local blur kernel. This offset value is determined by the local motion parameter set $\theta_{m}=\left\{v,v_{scan},A_{x},\omega_{x},\omega_{y}\right\}$.

Due to the uncertainty of motion parameter $\theta_{m}$, this paper employs image sharpness metrics as a feedback mechanism to perform precise local searches for optimal blur kernels within each partition, thereby optimizing the PSF. TDI blurred images exhibit distinct striped-like textures and concentrated high-frequency components predominantly aligned with the scanning direction. Based on this characteristic, three sharpness metrics are selected for coordinated estimation:

(26) $$\left\{\begin{array}{l} S_{TG}(f)=\sum_{i,j}\sqrt{G_{x}{\left(i,j\right)}^{2}+G_{y}{\left(i,j\right)}^{2}}\\ S_{Lap}(f)=\sum_{i,j}\left|f(i,j)*K_{Lap}\right|\\ S_{var}(f)=\frac{1}{pq}\sum_{i=1}^{p}\sum_{j=1}^{q}{\left(f(i,j)-\mu \right)}^{2}\\ S(f)=\alpha S_{TG}(f)+\beta S_{Lap}(f)+\gamma S_{var}(f) \end{array}\right.$$

where $S_{TG}(f)$ is the Tenengrad gradient metric that effectively quantifies edge energy, $S_{Lap}(f)$ is the Laplacian energy metric reflecting high-frequency components, and $S_{var}(f)$ is the variance metric indicating overall contrast within local regions, and S(f) is the sharpness evaluation index after the coordinated estimation. $K_{Lap}$ denotes the Laplacian kernel, where p and q denote the number of pixels in the row and column of the image, respectively; μ represents the mean gray value of all pixels in the local region; and α,β,γ are empirical weights.

For each partition $g_{i}$, construct a set of potential PSF candidates:

(27) $$\left\{h_{m}\left(\theta_{m,n}\right)\left|n=1,2,\ldots ,K\right.\right\}$$

Perform deconvolution on each potential PSF candidate:

(28) $${\overset{\wedge }{f}}_{i,k}=D\left(g_{i},h_{m}\left(\theta_{m,n}\right)\right)$$

where $D\left(\cdot \right)$ denotes the deconvolution operator, and ${\overset{\wedge }{f}}_{i,k}$ represents the restored partition image.

In practical deconvolution processes, directly solving the inverse problem without introducing constraints tends to cause severe noise amplification and ringing artifacts. To address this issue, this paper adopts total variation (TV) regularization as the deconvolution operator $D\left(\cdot \right)$. The ROF model proposed by Rudin et al. incorporates the L_1_-norm of the image gradient into a regularization framework, which effectively suppresses oscillations while preserving edge structures [27]. Based on this idea, TV regularization is introduced into the deconvolution process to enhance the stability and structural fidelity of the restored results, thereby improving the overall restoration quality. Its formulation is given as follows:

(29) $${\hat{f}}_{i,k}=\underset{f_{i,k}}{\arg\min}\left\{\frac{1}{2}{\left\|g_{i}-h_{m}\left(\theta_{m,n}\right)*f_{i,k}\right\|}_{2}^{2}+\mu \sum_{j=1}^{n^{2}}{\left\|\nabla f_{i,k}\right\|}_{2}\right\}$$

In the above equation, the first term is the data fidelity term, which constrains the restored image to remain as consistent as possible with the observed blurred image after undergoing the same blur degradation process. The second term is the regularization term, which imposes a L_1_-norm constraint on the image properties to suppress noise amplification and ringing artifacts generated during the deconvolution process, thereby producing results that are more consistent with natural images. Here, ${\left\|\cdot \right\|}_{2}^{2}$ denotes the squared L_2_-norm, ${\left\|\cdot \right\|}_{2}$ represents the vector second norm, $\sum_{j=1}^{n^{2}}{\left\|\cdot \right\|}_{2}$ denotes the total variation regularization term, $\nabla f_{i,k}$ represents the image gradient operator, and μ is the regularization parameter used to balance the data fidelity term and the regularization term.

In the time domain, the convolution operation is typically expressed as a matrix–vector multiplication. Therefore, the above equation can be rewritten as:

(30) $${\hat{F}}_{i,k}=\underset{f_{i,k}}{\arg\min}\left\{\frac{1}{2}{\left\|G_{i}-H_{m}\left(\theta_{m,n}\right)F_{i,k}\right\|}_{2}^{2}+\mu \sum_{j=1}^{n^{2}}{\left\|\nabla F_{i,k}\right\|}_{2}\right\}$$

In practical restoration, although the above TV regularization model can effectively improve image quality, the nonlinear nature of the L_1_-norm makes the optimization problem computationally demanding when solved directly, significantly increasing the overall runtime of the algorithm. To ensure restoration accuracy while reducing the computational cost of deconvolution, this paper employs the Split Bregman algorithm [28] to efficiently solve the aforementioned optimization problem.

The core idea of the Split Bregman method is to introduce auxiliary variables to decompose the original complex problem into several simpler subproblems that are easier to solve. Based on this decomposition, an iterative updating scheme is adopted to drive each variable to gradually converge to the global optimum.

After introducing the auxiliary variable d, the original optimization problem can be reformulated as follows:

(31) $${{\hat{F}}_{i,k}}^{\left(j+1\right)}=\underset{{\hat{F}}_{i,k},d^{\left(j\right)}}{\arg\min}\left\{\frac{1}{2}{\left\|G_{i}-H_{m}\left(\theta_{m,n}\right)F_{i,k}\right\|}_{2}^{2}+\mu \sum_{j=1}^{n^{2}}{\left\|d^{\left(j\right)}-\nabla F_{i,k}-b^{\left(j\right)}\right\|}_{2}\right\}$$

(32) $${{\hat{F}}_{i,k}}^{\left(j+1\right)}=\underset{{\hat{F}}_{i,k},d^{\left(j\right)}}{\arg\min}\left\{\frac{1}{2}{\left\|G_{i}-H_{m}\left(\theta_{m,n}\right)F_{i,k}\right\|}_{2}^{2}+\sum_{j=1}^{n^{2}}\left[{\left\|d^{\left(j\right)}\right\|}_{2}+\frac{\mu }{2}{\left\|d^{\left(j\right)}-\nabla F_{i,k}-b^{\left(j\right)}\right\|}_{2}^{2}\right]\right\}$$

where $d^{\left(j\right)}=\nabla F_{i,k}$ and $b^{\left(j\right)}=d^{\left(j\right)}-\nabla F_{i,k}$ are both auxiliary parameters used in the split Bregman algorithm.

The above formulation can be decomposed into two subproblems that are solved iteratively: Subproblem ${\hat{F}}_{i,k}$ and Subproblem $d^{\left(j\right)}$.

With the value of $d^{\left(j\right)}$ fixed, Equation (32) is reduced to a minimization problem with respect to ${\hat{F}}_{i,k}$. The variable ${\hat{F}}_{i,k}$ can be updated by solving a quadratic least-squares problem, which is computed using the Gauss–Seidel iteration method. The specific iterative formula is given as follows:

(33) $$\begin{array}{ll} {{\hat{F}}_{i,k}}^{\left(j+1\right)}= & \frac{1}{4\mu +1}\left[{\left(H_{m}{\left(\theta_{m,n}\right)}^{T}\right)}_{i,k}+\mu \left({{\hat{F}}_{i-1,k}}^{\left(j+1\right)}+{{\hat{F}}_{i+1,k}}^{\left(j\right)}+{{\hat{F}}_{i,k-1}}^{\left(j+1\right)}+{{\hat{F}}_{i,k+1}}^{\left(j\right)}\right)\right.\\ & \left.+\mu \left(d_{i-1,k}^{\left(j\right)}-d_{i+1,k}^{\left(j\right)}+d_{i,k-1}^{\left(j\right)}-d_{i-1,k+1}^{\left(j\right)}\right)-\mu \left(b_{i-1,k}^{\left(j\right)}-b_{i+1,k}^{\left(j\right)}+b_{i,k-1}^{\left(j\right)}-b_{i-1,k+1}^{\left(j\right)}\right)\right] \end{array}$$

With the value of ${\hat{F}}_{i,k}$ fixed, Equation (32) is reduced to a minimization problem with respect to $d^{\left(j\right)}$. The problem is solved according to the two-dimensional shrinkage theory, and the resulting iterative formula is given as follows:

(34) $$d^{\left(j\right)}=\max\left[{\left\|\nabla F_{i,k}+b^{\left(j\right)}\right\|}_{2}-\frac{1}{\mu }\right]\frac{\nabla F_{i,k}+b^{\left(j\right)}}{{\left\|\nabla F_{i,k}+b^{\left(j\right)}\right\|}_{2}}$$

Based on the above solution, the value of $b^{\left(j\right)}$ can be updated as follows:

(35) $$b^{\left(j+1\right)}=d^{\left(j+1\right)}-\nabla {F_{i,k}}^{\left(j+1\right)}$$

The sharpness index is calculated using a sharpness evaluation function, and the optimal PSF is iteratively selected as the estimate, ${\hat{f}}_{n,k}$ represents the restored partitioned image when the optimal PSF is selected as the estimation value:

(36) $$n^{*}={\mathrm{argmax}}_{n}S\left({\hat{f}}_{n,k}\right)$$

After selecting the optimal PSF estimate for each partition and completing the deconvolution restoration of the partitioned images, all locally restored images must be stitched and fused to obtain a final restored image with continuous structure, consistent brightness, and clear details. Since different PSFs are used for deconvolution across regions, direct stitching would cause noticeable brightness jumps and structural discontinuities at boundary areas. Therefore, this paper employs a weighted average fusion method to achieve natural transitions between local regions and overall structural consistency.

### 3.2. Image Quality Evaluation Algorithm

Common image quality evaluation metrics include Structural Similarity Index (SSIM) and Peak Signal-to-Noise Ratio (PSNR) [29]. Their calculation formulas are as follows:

(37) $$\begin{array}{l} SSIM=\frac{\left(2\mu_{x}\mu_{y}+C_{1}\right)\left(2\sigma_{\mathrm{xy}}+C_{2}\right)}{\left({\mu_{x}}^{2}+{\mu_{y}}^{2}+C_{1}\right)\left({\sigma_{x}}^{2}+{\sigma_{y}}^{2}+C_{2}\right)}\\ PSNR=10\times \mathrm{lg}\left(\frac{MaxValue^{2}}{MSE}\right) \end{array}$$

where $\mu_{x}$ and $\mu_{y}$ represent the mean values of image x and image y within their respective local regions, $\sigma_{x}$ and $\sigma_{y}$ denote the standard deviations of image x and image y, while $\sigma_{xy}$ denotes the covariance between the two images. MaxValue is the maximum pixel value achievable in the image, and MSE is the mean squared error between the two images. Generally, the closer the SSIM value is to 1 and the larger the PSNR value, the better the image quality.

In practical scenarios where ideal reference images are unavailable, no-reference image quality assessment metrics are commonly employed to evaluate the restoration performance of algorithms under real degradation conditions. Natural Image Quality Evaluator (NIQE) [30] and Perception-based Image Quality Evaluator (PIQE) [31] are two widely used no-reference quality assessment metrics. Their formulations are given as follows:

(38) $$\begin{array}{ll} NIQE & =D(v_{1},v_{2},\sum_{1},\sum_{2})=\sqrt{{\left(v_{1}-v_{2}\right)}^{T}{\left(\frac{\sum_{1}+\sum_{2}}{2}\right)}^{-1}\left(v_{1}-v_{2}\right)}\\ PIQE & =\frac{\left(\sum_{k=1}^{N_{SA}}D_{sk}\right)+C_{1}}{\left(N_{SA}+C_{1}\right)} \end{array}$$

where $v_{1},v_{2}$ and $\sum_{1},\sum_{2}$ are the mean vectors and covariance matrices of the natural MVG model and the distorted image’s MVG model. $N_{SA}$ is the number of Spatially Active blocks in a given image. $D_{sk}$ represents the local activity level of the k-th distortion block. $C_{1}$ is a positive constant.

## 4. Experiments and Analysis

### 4.1. Simulated Blurred Image Restoration Experiment

In this study, to validate the effectiveness of the proposed algorithm in addressing TDI imaging blur caused by UAV platform vibration and velocity mismatch, simulation experiments were first conducted on a public dataset, VDD [32]. A high-resolution image, DJI0667, from the VDD test set was selected as the reference. The algorithm was then used to synthesize blurred images, simulating the blurring effects that may occur during actual flight in TDI imaging. TDI imaging relies on the accumulation of charge over multiple stages, rather than a single exposure. When the charge transfer speed does not match the target’s relative motion speed, TDI produces motion blur, with a small, fixed displacement occurring at each integration stage. Since the line-scan period of a TDI camera is extremely short, the speed difference can be considered constant across adjacent lines. Physically, this constant speed mismatch manifests as a linear smear along the motion direction, resulting in a straight-line displacement trajectory across neighboring rows. Based on this physical principle, we simulate TDI motion blur using a linear motion kernel convolution model with random directions and lengths, and we additionally add Gaussian noise to better approximate the TDI imaging process.

**Figure 5:**
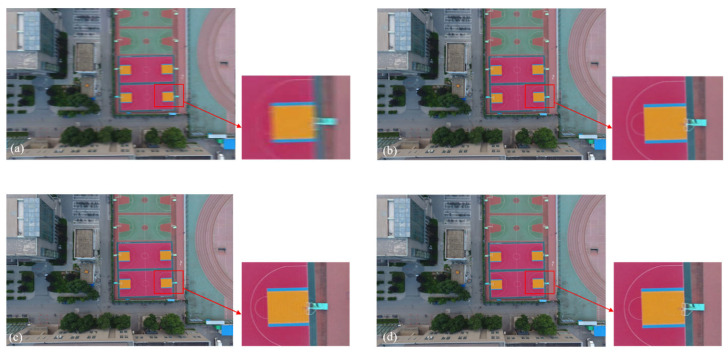
Synthetic blurred image and restoration results: (**a**) Blurred image of DJI0667; (**b**) Algorithm proposed in reference [33]; (**c**) Algorithm proposed in reference [34]; (**d**) Algorithm proposed in this paper.

Figure 5 shows the motion blur model-synthesized images during simulated TDI imaging and the restoration results of different algorithms. The proposed algorithm in this paper is compared with the algorithms presented in [33,34]. The restoration results from the algorithm in [33] show insufficient detail recovery, and the visual quality needs to be improved. While the algorithm in [34] improves overall image quality to some extent, residual noise persists in certain regions, and the restored images exhibit slight artifacts. In contrast, the proposed algorithm in this paper effectively reduces ringing effects, eliminates artifacts, and enhances the overall image quality.

To validate the universality of the proposed algorithm in this paper, five additional images were selected from the VDD dataset, encompassing road vehicles, buildings, and natural landscapes. These images were processed using the same algorithm as in Figure 5 to generate blurred versions. Therefore, a total of six test samples were used for quantitative evaluation. Subsequently, the images were restored using the algorithms in Reference [34], as well as the algorithm proposed in this paper, and the restoration results were evaluated using SSIM and PSNR. The numerical results are recorded in Table 1. The results show that the proposed algorithm in this paper effectively restores image details and structure, enhancing image quality. Compared to the algorithm in [33] and the algorithm in [34], the proposed algorithm in this paper achieves approximately 2 dB higher PSNR and about 0.03 higher SSIM. This demonstrates the significant advantages of the proposed algorithm in this paper in TDI blurred image restoration.

### 4.2. Experimental System

**Figure 6:**
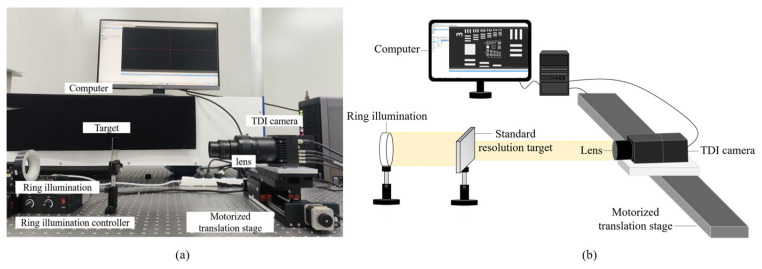
TDI Imaging System: (**a**) System physical diagram; (**b**) System schematic diagram.

To validate the algorithm’s feasibility in real imaging systems, this paper constructs a TDI camera imaging system. As shown in Figure 6, the system comprises a TDI line-scan camera, a dual telecentric adjustable-focus objective lens, an electric displacement stage, a target, and a control computer. The TDI camera model is PH9KCXP13-530KT256, featuring a pixel size of 5 μm and supporting up to 256-stage charge integration. The light source model is AI-RL-90-60W, a ring light. The objective lens model is LS1610A with a magnification of 1×. The motion platform operates at a maximum speed of 20 mm/s, with motor controllers enabling precise adjustment of push-broom scanning speeds to simulate varying degrees of blur. The experimental target utilizes the standard resolution target.

**Figure 7:**
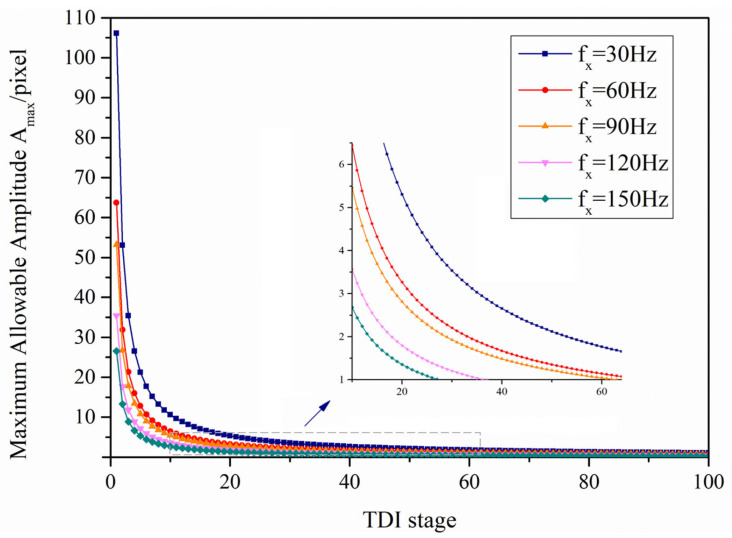
Relationship between TDI integral series and maximum allowable amplitude at different vibration frequencies.

Based on the platform vibration analysis principles in Section 3.1 and the MTF calculation formula for the TDI imaging system, the imaging quality of the system is jointly governed by the TDI stages, vibration frequency, and vibration amplitude. For the TDI imaging system constructed, the maximum allowable vibration amplitude under different vibration frequencies and TDI stages can be numerically simulated and vibration amplitude. As shown in Figure 7, at the same vibration frequency, the maximum allowable amplitude decreases with increasing numbers of TDI stages. At the same TDI stage, the maximum allowable amplitude increases as the vibration frequency decreases.

The primary sources of blur in UAV imaging include linear vibration image shifts $\delta_{l}$ caused by axial displacement of the airframe and angular vibration image shifts $\delta_{a}$ due to UAV pitch or roll. These two types of image shifts are mainly influenced by platform altitude and attitude vibration. Linear vibration image shift is primarily influenced by platform altitude. According to the velocity-matching relationship and imaging model derived in Section 2, platform altitude and image shift velocity are inversely related, making UAV low-altitude flight conditions highly sensitive to vertical stability disturbances. Taking the DJI Mini SE as an example, to balance stabilization accuracy and imaging resolution, the flight altitude should be set between 20 m and 120 m. The angular vibration image shift can be represented as $\delta_{a}=f\cdot \tan\left(\Delta \theta \right)\approx f\cdot \Delta \theta$, where the maximum allowable angular deviation is $\Delta \theta \leq \frac{\delta_{a}}{f}$. For the DJI Mini SE, the camera lens has an equivalent focal length of 24 mm and a physical focal length of 4.5 mm. To limit the image shift to within a single pixel, the theoretically permissible maximum angular deviation is $\Delta \theta \leq \frac{5\;\mu m}{f}=\frac{5\;\mu m}{4.3\;\mathrm{mm}}\approx 0.0012rad\approx 0.069{}^{\circ}$. On actual UAV platforms, the gimbal stabilization capabilities can reach ±0.01°, which satisfies the angular deviation tolerance for angular vibration image shifts. Based on the gimbal anti-vibration data from DJI drones and the comparative analysis of the TDI stages and maximum allowable amplitude under different tremor frequencies through simulation results, it can be concluded that the existing anti-vibration capability of the UAV platform can control the amplitude within the required limits.

**Figure 8:**
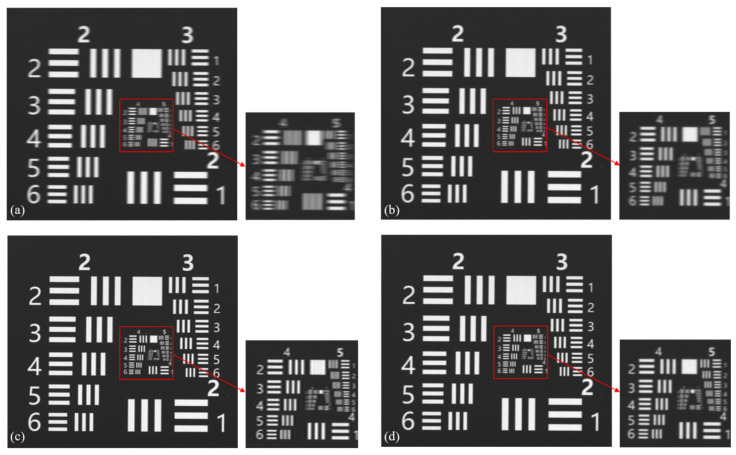
Blurred image of resolution target captured by TDI and restoration results: (**a**) Blurred image; (**b**) Algorithm proposed in reference [33]; (**c**) Algorithm proposed in reference [34]; (**d**) Algorithm proposed in this paper.

Figure 8 shows the blurred resolution chart images captured using a TDI imaging system with a 10% speed mismatch, along with the restoration results from different algorithms. The proposed algorithm in this paper is compared with the methods described in [34], and the restoration results are evaluated using NIQE and PIQE. The results are presented in Table 2. From the data in Table 2, it can be seen that the algorithm in this paper achieves the optimal values in both indicators. Specifically, the NIQE has decreased to 4.7558, and the PIQE has significantly dropped to 50.6011, which represents an improvement of approximately 25% and 34%, respectively, compared to the blurred images. By combining the visual analysis in Figure 8, it can be observed that although Figure 8b achieves some improvement in image sharpness, its detail recovery capability is poor, and the restoration results for the fourth resolution group are unsatisfactory. Figure 8c shows a noticeable improvement compared to Figure 8b, but magnification reveals significant edge sharpening and artifacts in the image. In contrast, the proposed algorithm in this paper constructs a TDI velocity mismatch degradation physical model and employs partitioned iterative PSF estimation. This approach yields a more precise PSF, avoids artifact generation, and achieves significant improvements in sharpness and overall image quality.

### 4.3. Real Blurred Image Restoration Experiment

**Figure 9:**
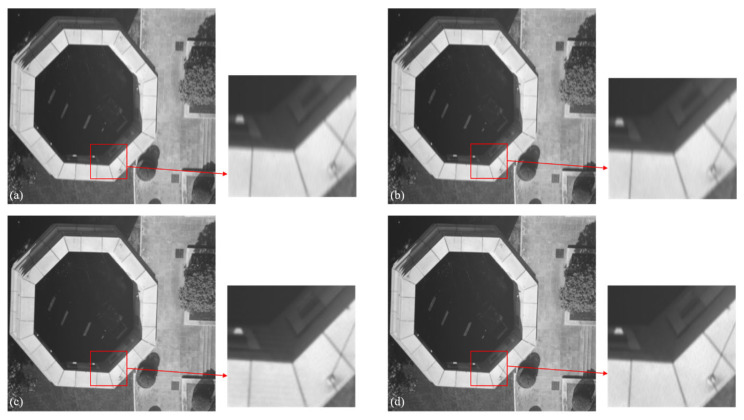
Blurred image of actual campus roads and tower tops, along with their restoration results: (**a**) Blurred image; (**b**) Algorithm proposed in reference [33]; (**c**) Algorithm proposed in reference [34]; (**d**) Algorithm proposed in this paper.

To evaluate the performance of the algorithm in real-world scenarios, we collected data using a DJI Mini SE drone over two types of scenes: campus buildings and roads. During the experiments, the drone maintained a flight altitude of 40–50 m, with the TDI camera set to 128 integration stages and a line rate of approximately 4800 Hz. Due to the dynamic nature of the flight environment, the captured images in both scene types exhibited varying degrees of velocity-mismatch blur. Subsequently, the proposed algorithm in this paper and methods from [34] were applied to restore the blurred images. Combined with the quantitative evaluation in Table 3, the proposed algorithm outperforms the comparison methods in terms of both NIQE and PIQE, objectively validating the enhancement of the restored images in naturalness and perceptual quality. In terms of visual effects, Figure 9b demonstrates some detail recovery, but lines in the enlarged region remain significantly blurred, indicating limited deblurring effectiveness; Figure 9c reveals more detail recovery in the enlarged area, yet the restored image exhibits artifacts and ringing effects. In contrast, the enlarged region of the image restored by the proposed algorithm in this paper remains relatively clear, with well-controlled noise and superior image quality.

**Figure 10:**
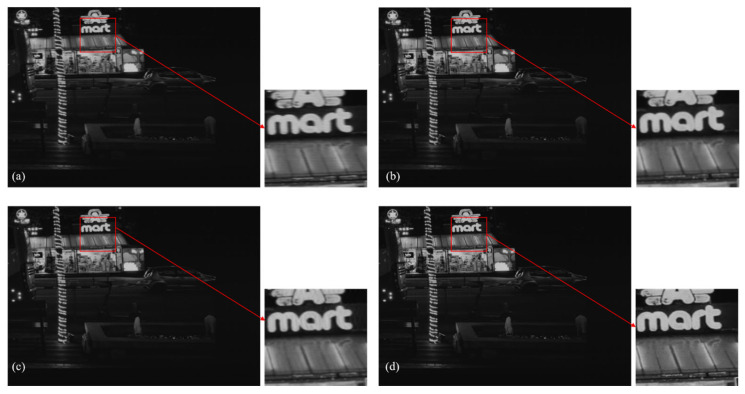
Blurred image of a low-light scene and restoration results: (**a**) Blurred image; (**b**) Algorithm proposed in reference [33]; (**c**) Algorithm proposed in reference [34]; (**d**) Algorithm proposed in this paper.

To further validate the performance of the proposed algorithm in this paper under low-light conditions, real-world low-light scenes were captured by UAVs during nighttime operations. Nighttime imaging is constrained by natural illumination, resulting in images with low signal-to-noise ratios. Although TDI imaging systems can enhance signal strength to some extent through multi-stage charge accumulation, the increased exposure time also makes them more susceptible to factors such as speed mismatch. This leads to blurred image details and structural distortion. Figure 10 and Table 4, respectively, show the low-light night TDI blurred images and the results and quantitative evaluation indicators after being restored by different algorithms. From Table 4, it can be seen that the algorithm in this paper performs optimally in terms of NIQE and PIQE indicators. Combined with the visual effect analysis in Figure 10, it can be observed that the algorithm proposed in [33] achieves some success in enhancing image clarity but remains insufficient in restoring fine details, resulting in limited restoration effectiveness. Compared to the algorithm in [33], the one in [34] offers improved restoration with higher image clarity. However, magnified regions reveal persistent artifacts and ringing effects when processing nighttime images. The algorithm proposed in this paper delivers superior overall image quality, featuring sharp edges and clear icon content, thereby achieving enhanced restoration performance. In summary, compared to other restoration algorithms, the proposed method demonstrates superior performance in addressing TDI imaging blur caused by combined factors such as UAV platform vibration and velocity mismatch.

## 5. Conclusions

This paper addresses the motion blur problem caused by the coupled effects of platform vibration and velocity mismatch during TDI push-broom imaging on UAVs. It proposes a TDI image restoration algorithm based on region-wise PSF estimation guided by an optimal-sharpness criterion. Through an in-depth analysis of TDI image degradation mechanisms, this paper constructs a physics-consistent TDI image degradation model tailored for UAV platforms. This model explicitly accounts for the coupled effects of platform vibration and velocity mismatch, emphasizing the row-dependent and non-stationary characteristics of the blur kernel caused by the charge step-by-step accumulation inherent to TDI image sensors. It formulates a degradation model that accurately describes how image shift errors propagate and progressively accumulate across successive charge transfer cycles. Building upon this foundation, the study transforms the complex spatial blur kernel problem into a partitioned optimal PSF search and adaptive selection framework. It employs a sharpness evaluation function composed of three metrics, namely Tenengrad gradient, Laplacian energy, and variance, as a feedback mechanism to iteratively select the optimal PSF estimate. Comparative experiments were conducted on blurred images simulated by convolving with randomly oriented linear motion kernels and on actual blurred images captured by UAV TDI. The results demonstrate that the proposed algorithm not only restores TDI blurred images but also effectively suppresses ringing effects and artifacts, significantly enhancing image quality.

In addition, this paper analyzes the influence of velocity mismatch in UAV-based TDI push-broom imaging. When the push-broom scanning direction is inconsistent with the UAV flight direction, the equivalent image motion velocity changes, thereby introducing additional velocity mismatch errors. In practical applications, such errors can be mitigated by appropriately adjusting the UAV flight direction to align it with the push-broom scanning direction, thus reducing the velocity mismatch induced by directional inconsistency. Future work will focus on investigating adaptive and dynamically adjustable TDI stage control mechanisms, further optimizing the partitioning strategy to reduce computational complexity, and exploring the application of the proposed method in more complex motion scenarios, with the aim of further improving the imaging quality of TDI systems in complex dynamic environments.

## Figures and Tables

**Table 1:** Quantitative evaluation metrics for the restoration results of six synthetic blurred images.

Picture	Algorithm in Reference [33]		Algorithm in Reference [34]		Proposed Algorithm in This Paper	
	SSIM	PSNR/dB	SSIM	PSNR/dB	SSIM	PSNR/dB
000041	0.8232	27.33	0.8374	28.17	0.8761	28.59
01393041	0.8169	27.78	0.8710	29.21	0.8966	31.03
DJI0139	0.7502	27.96	0.8279	29.67	0.8477	30.87
DJI00584	0.7292	27.97	0.8454	30.70	0.8615	31.86
DJI00587	0.7546	26.89	0.8409	29.10	0.8577	29.62
DJI0667	0.8758	31.54	0.8982	32.54	0.9173	33.34

**Table 2:** Quantitative evaluation metrics for the restoration results of the target capture.

Metrics	Blurred Image	Algorithm Proposed in Reference [33]	Algorithm Proposed in Reference [34]	Algorithm Proposed in This Paper
NIQE	6.3562	5.8490	5.1696	4.7558
PIQE	76.9843	58.2723	57.9898	50.6011

**Table 3:** Quantitative evaluation metrics for the restoration results of the actual campus roads and tower tops.

Metrics	Blurred Image	Algorithm Proposed in Reference [33]	Algorithm Proposed in Reference [34]	Algorithm Proposed in This Paper
NIQE	5.2890	4.3173	4.2890	4.0931
PIQE	64.8736	55.4406	52.1952	45.1238

**Table 4:** Quantitative evaluation metrics for the restoration results of a low-light scene.

Metrics	Blurred Image	Algorithm Proposed in Reference [33]	Algorithm Proposed in Reference [34]	Algorithm Proposed in This Paper
NIQE	6.1665	5.2074	5.0848	4.6285
PIQE	76.5552	58.8737	55.0295	47.4418

## Data Availability

The data analyzed in this study are all contained within the article. For additional information, please contact the corresponding author.
